# Experiments and Modeling of Machined Spring Rotary Actuators with Shape Memory Alloys

**DOI:** 10.3390/ma15196674

**Published:** 2022-09-26

**Authors:** Tiegang Chen, Yuhang Zhang, Shengbin Qiu, Jun Jiang, Qiang Zhang, Xiaoyong Zhang

**Affiliations:** 1 School of Energy and Power Engineering, Beihang University, Beijing 100191, China; 2 Shaanxi Qianshan Avionics Co., Ltd., Xi’an 710065, China; 3 Beijing Institute of Control Engineering, Beijing 100190, China; 4 Collaborative Innovation Center of Advanced Aero-Engine, Beijing 100191, China; 5 National Key Laboratory of Science and Technology on Aero-Engine Aero-Thermodynamics, Beijing 100191, China; 6 Beijing Key Laboratory of Aero Engine Structure and Strength, Beijing 100191, China

**Keywords:** machined spring, rotary actuator, shape memory alloy, rectangular cross-section, NiTi

## Abstract

This paper presents a novel rotary actuator using an NiTi shape memory alloy machined spring (SMAMS). An analytical model is put forward to describe the relationship between the twist angle and temperature of SMAMSs under different applied torques. Following that, a numerical model is developed to analyze the stress distributions and twist angle-torque responses of the SMAMS, tube, and spring of the circular cross-section. Thus, the advantages of the SMAMS over the other two rotary actuators are obtained. Moreover, experiments with SMAMSs are conducted to validate these models and study their mechanical responses. Results show that the SMAMS can be designed to have a larger twist angle than the cylindrical-type rotary actuators and to bear a larger torque than the wire-based-type rotary actuators, provided that the inner and outer diameter remains unchanged. Specifically, the maximum actuating twist angle of SMAMSs reaches 278.5°, and their maximum actuating torque is 0.312 N·m. The maximum two-way twist angle of SMAMSs reaches 171° at the pre-applied torque of 0.12 N·m. Moreover, the geometry is found to have a significant influence on the actuating capacity of SMAMSs. When the moments of inertia of SMAMS are 0.82 and 4.69, the corresponding torsion angles are 185.3° and 29.8°, respectively. In general, the SMAMSs with a larger moment of inertia can withstand a larger load. This work fills the gap between wire-based-type rotary actuators and cylindrical-type rotary actuators and is expected to expand the use for SMAs in the rotary actuator.

## 1. Introduction

Recently, the rotary actuator has attracted extensive attention due to its significant applications in space mechanisms. Conventional rotary actuators are mainly hydraulic rotary actuators and electrical motors [1], which can accurately output arbitrary angles to meet actuating requirements. However, the former is big and heavy, and its structure is complex; the latter needs a gearbox to generate high torque. Therefore, they are not suitable for use in a demanding space environment. To solve these problems, shape memory alloy (SMA) actuators have been proposed in recent years. SMAs show unique properties at high and low temperatures. After being deformed at a low temperature, they can return to their shape at high temperature because they can remember their high-temperature shape [2,3]. Due to the unique actuating mechanism of material transformation, they can be small, light and simple. These advantages make SMA actuators possible alternatives to conventional actuators. Because of the development of shape memory alloy machining technology [4,5] and actuator design theory [6], shape memory alloy actuators have been successfully applied in the biomedical [2], aerospace [7], engineering [8] and other fields.

SMA rotary actuators have two forms. One is that the linear motion of SMA components is transformed into rotational motion. The other is that the SMA component directly generates rotational motion [9,10]. In the former method, an SMA straight wire [11,12,13] or SMA spring [14] is heated and contracted, producing a linear motion that actuates the mechanism to generate a continuous or discontinuous rotation. However, compared to the latter method, this method relies on a conversion mechanism that is complex and less efficient. The SMA components that generate rotational motion directly can be classified as the wire-based type (wires [15,16], springs of circular cross-section [17,18,19]), the plate-based type [20,21,22,23,24] or the cylindrical type (bars [25] or tubes [26,27,28,29,30,31,32,33]) in terms of configuration. For example, Yan [18] designed a rotary actuator using an SMA spring with a circular cross-section whose maximum actuating twist angle is 340° while the maximum actuating torque is 12 × 10^−6^ N·m. Paik [21] proposed an SMA plate whose maximum actuating twist angle is 180°, while the maximum actuating torque is 4 × 10^−3^ N·m. Benafan [28] proposed a novel SMA tube with high torque capability whose maximum actuating torque is 1400 N·m, but its maximum actuating twist angle is only 25°. Due to their structural characteristics, the wire-based type and the plate-based type actuators have a larger actuating twist angle but a smaller actuating torque, while cylindrical-type actuators have a smaller actuating twist angle but a larger actuating torque. If large actuating twist angles and actuation torques are required at the same time, the rotary actuator can only be achieved by significant resizing. For example, a large actuating twist angle can only be obtained by drastically reducing the diameter or increasing the length of the SMA tube. However, sometimes it is impossible to change the diameter or increase the length because of dimensional constraints. To solve this problem, we propose a novel SMA rotary actuator whose actuating twist angle is larger than that of the cylindrical-type rotary actuators and whose actuating torque is larger than that of the wire-based-type rotary actuators, provided that the inner and outer diameter of the rotary actuator remain unchanged.

In this article, we present a novel rotary actuator using a NiTi shape memory alloy machined spring (SMAMS). Compared with traditional hydraulic rotary actuators and electric motors, the SMAMS is space-saving, light and simple in structure. Compared with other types of SMA rotary actuators, SMAMS can be designed to have a larger twist angle than cylindrical-type rotary actuators and to bear a larger torque than the wire-based-type rotary actuators, with the inner and outer diameter remaining unchanged. This rotary actuator fills the gap between wire-based-type rotary actuators and cylindrical-type rotary actuators.

This article is organized as follows. The analytical model of the SMAMS is briefly introduced. This model can be used to guide the selection of the appropriate parameters for the SMAMS. Following that, a numerical model is developed to examine the stress distributions and twist angle-torque responses of the spring of the circular cross-section, the SMAMS and the tube. Thus, the advantages of the SMAMS over the spring of the circular cross-section and the tube are obtained. Moreover, experiments on the SMAMSs are conducted to study their mechanical responses. Finally, conclusions are discussed.

## 2. Analytical Model of the SMAMS

**Figure 1:**
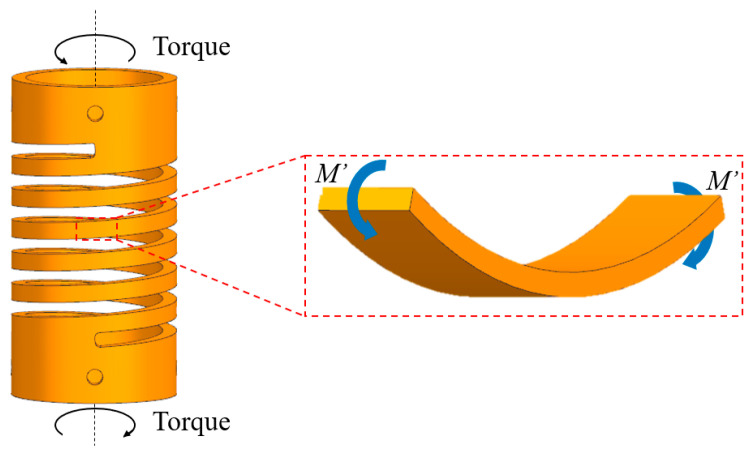
The SMAMS under the torque.

The selected SMA are binary Ni_50_Ti_50_ (at.%) alloys. During the actuation, we assume that the spring diameter is constant. When the spring is subjected to torque, the coil section is subjected to a bending moment, as shown in Figure 1. The SMAMS contains both the coil section and end piece. The coil section, called flexure, provides elasticity. The end pieces are much stiffer compared to the flexure and are used as an interface with the adjoining structure. Therefore, SMAMSs can be used as connectors and actuators.

For a torsion spring, stress is correlated with torque by Shigley [34]:

(1) $$\sigma =K\cdot \frac{M}{\left(I/c\right)}$$

where *M* is the torque, *I*/*c* is the section modulus and *K* is the stress concentration factor approximately equal to one.

The maximum normal strain in the beam with a rectangular cross-section is given by:

(2) $$\varepsilon =\frac{\theta \cdot D}{2L_{s}}$$

where *D*, *θ* and *L*_s_ are the spring mean diameter, angle of the torsion spring and length of the SMA beam, respectively. The *L*_s_ is given by:

(3) $$L_{s}=N\pi D$$

where *N* is the number of active coils.

The volume fraction of martensite can be obtained from the model of Brinson [35,36] and Liang [37], as follows:

(4) $$\sigma -\sigma_{0}=D\left(\xi \right)\cdot \varepsilon -D\left(\xi_{0}\right)\cdot \varepsilon_{0}+\Omega \left(\xi \right)\cdot \xi_{s}-\Omega \left(\xi_{0}\right)\cdot \xi_{s0}+\Theta \left(T-T_{0}\right)$$

*M*→*A*:

(5) $$\xi =\frac{\xi_{0}}{2}\left\{\cos\left[a_{A}\left(T-A_{s}-\frac{\sigma }{C_{A}}\right)\right]+1\right\}$$

(6) $$a_{A}=\pi /\left(A_{f}-A_{s}\right)$$

(7) $$D=D_{a}+\xi \cdot \left(D_{m}-D_{a}\right)$$

where *T*, *σ* and *ε* are the temperatures, applied stress and strain, respectively. Ω is the transformation tensor, and Θ is related to the thermal expansion coefficient of SMA material. *D*_*m*_ is the modulus value of martensite while *D*_a_ is that of austenite for the SMA. *ξ* denotes the martensite fraction. *ξ*_0_ is the fraction of martensite in the initial state. *C*_A_ is a material property that describes the relationship between temperature and induced transformation. Table 1 shows the parameters of SMA material properties.

**Figure 2:**
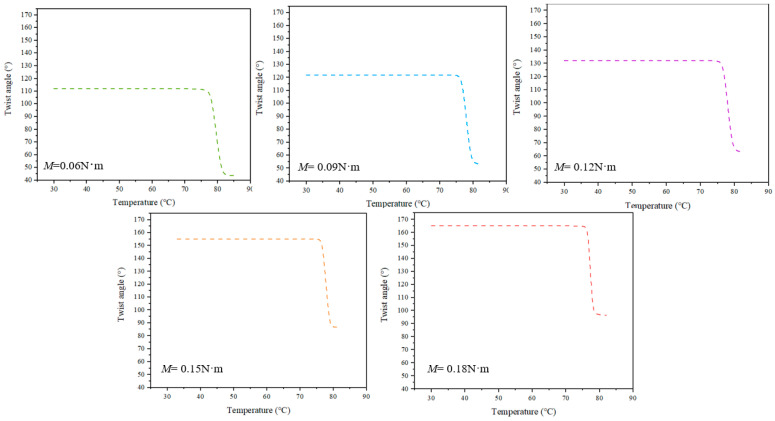
The analytical results of the twist angle–temperature curves of the SMAMS with different applied torques.

Figure 2 shows the twist angle–temperature curves of the SMAMS with different torques, which were calculated by Equations (1)–(7).

**Figure 3:**
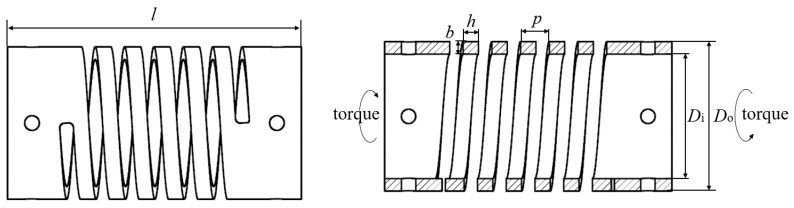
Schematics of the SMAMS.

Four different sizes of SMAMSs were selected to investigate the effect of geometry on actuating capability. The outer diameters of SMAMSs S1, S2 and S3 are *D*_o_ = 30 mm, and their other dimensions are *D*_i_ = 26.6 mm, *b* = 1.6 mm and *n* = 5. Their cross-sections parallel to the SMAMS axis are *h* = 2.4, 3.2 and 4.8 mm, respectively. The outer diameters of SMAMSs S4 are *D*_o_ = 31.2 mm, and its other dimensions are *D*_i_ = 26.6 mm, *b* = 1.6 mm, *n* = 5 and *h* = 3.2 mm. The overall length and pitch of SMAMSs are 60 mm and 6 mm, respectively. These geometric parameters are shown in Table 2. Figure 3 shows a schematic of an SMAMS geometry.

## 3. Finite Element Analysis of the SMAMS

To analyze the advantages of the SMAMS over the spring of the circular cross-section and the tube, a numerical simulation was conducted. We chose to study the coil section that is relevant to the actuation. The SMAMS S4 and the tube have the same wall thickness of 2.6 mm, and the wire diameter of the spring of the circular cross-section is 2.6 mm. The torque generated by the weight was applied to one end of the coil section in the form of the angular load causing the coil to twist. Meanwhile, a fixed constraint was applied to the other. The constitutive model of the material was proposed in Lagoudas [38]. In the commercial software package ABAQUS/Standard (2019), triangular, linear 3D stress elements (type C3D8) and static, general steps were used to analyze the coil.

**Figure 4:**
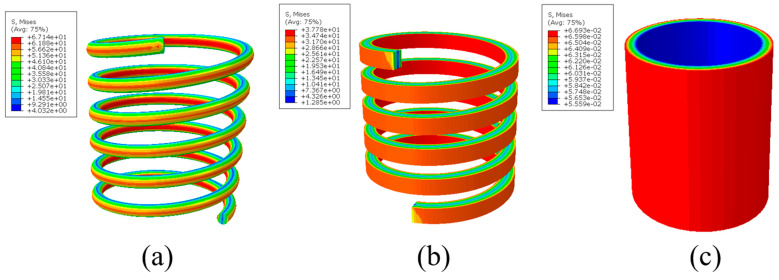
The Von Mises stress distributions of (**a**) the spring of the circular cross-section, (**b**) the SMAMS and (**c**) the tube under the torque of 0.1 N·m.

Figure 4 shows the stress distributions of the spring of the circular cross-section, the SMAMS S4 and the tube under a torque of 0.1 N·m. We find that the SMAMS and the spring of the circular cross-section have similar stress distributions, with a region of high stress on the inner and outer surface and a region of low stress on the middle surface, which confirms the above analysis that the coil section bears the bending moment. Different from them, the region of high stress of the tube lies on the outer surface and the region of low stress lies on the inner surface. This means that the tube and the spring have different actuating mechanisms. Compared to the tube, the actuating mechanism of the SMAMS is more similar to that of the spring of the circular cross-section.

**Figure 5:**
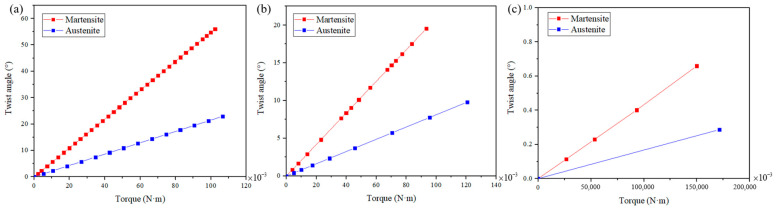
The twist angle-torque responses of (**a**) the spring of the circular cross-section, (**b**) the SMAMS S4 and (**c**) the tube.

Figure 5 shows the twist angle-torque responses of these three actuators. The red line in the figure represents martensite, and the blue line represents austenite. As can be seen from this figure, with the same torque, the actuating twist angle of the SMAMS is larger than that of the tube and smaller than that of the circular section spring; Instead, with the same rotation angle, the bearing capacity of the SMAMS is smaller than that of the tube and larger than that of the circular section spring, provided that the inner and outer diameter remains unchanged; the SMAMS can be designed to have a larger twist angle than the tube and to bear a larger torque than the spring of the circular cross-section, which provides a new option for a rotary actuator between the tube and the spring of the circular cross-section.

It is worth noting that only the elastic deformation is involved in finite element analysis, and the martensite transformation is not considered. On the one hand, the properties of different structures can be better compared through elastic deformation than martensite transformation. On the other hand, the martensite transformation of the SMAMS does not converge easily in the finite element analysis, which can easily cause incorrect results. Therefore, only elastic deformation is involved here.

## 4. Experimental Validation

**Figure 6:**
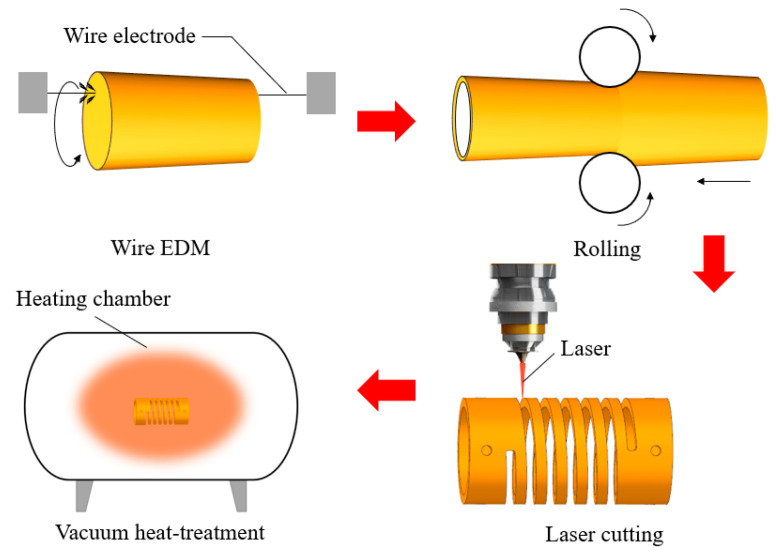
Fabricating process of the SMAMS.

A Ni_50_Ti_50_ alloy was produced by vacuum induction skull melting, and then it was processed into tubes using wire EDM and rolling technology. In the next step, tubes were cut by the laser-cut machine (HKCF-150, Beijing Huike Laser Technology Co., Ltd., Beijing, China), and the resulting SMAMSs are subjected to a vacuum heat treatment of 1 h at 500 °C in a vacuum heat-treatment furnace, as shown in Figure 6. Then they are air-cooled.

### Thermomechanical Response

**Figure 7:**
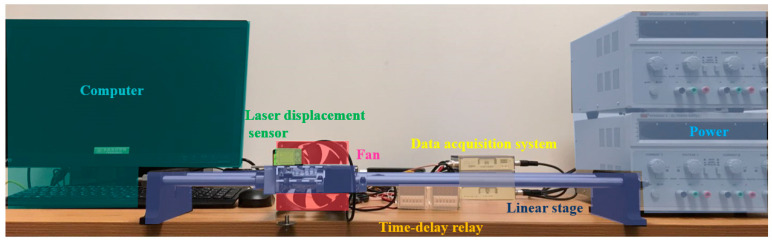
Thermomechanical test setup.

An experimental setup was developed to test the SMAMS’s thermomechanical responses. This setup consists of a laser displacement sensor, data acquisition system, time-delay relay, linear stage, computer, power, fan, thermocouple, heater and load, as shown in Figure 7.

**Figure 8:**
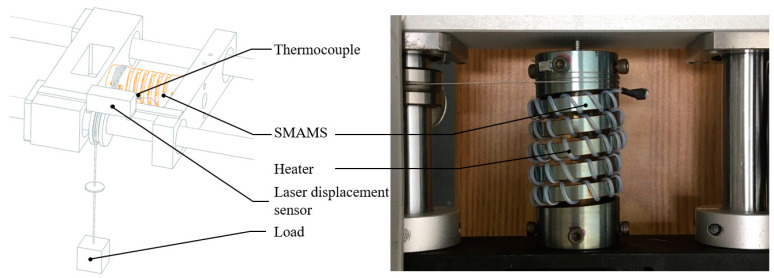
Detailed experimental setup for the thermomechanical test.

The SMAMS is mounted into the experimental setup. The temperature is monitored by the type-K thermocouples, which are stuck on the middle of the SMAMS. The displacement is monitored by the laser displacement sensor and then processed into angles in the computer. The torque produced by the load is acting on the SMAMS, as shown in Figure 8. When actuated, power is supplied to the heater, and the SMAMS is heated to rotate. When not actuated, power is supplied to the fan instead of the heater, and the SMAMS cools and returns under the torque. The time delay relay is used to control the time of heating and cooling.

**Figure 9:**
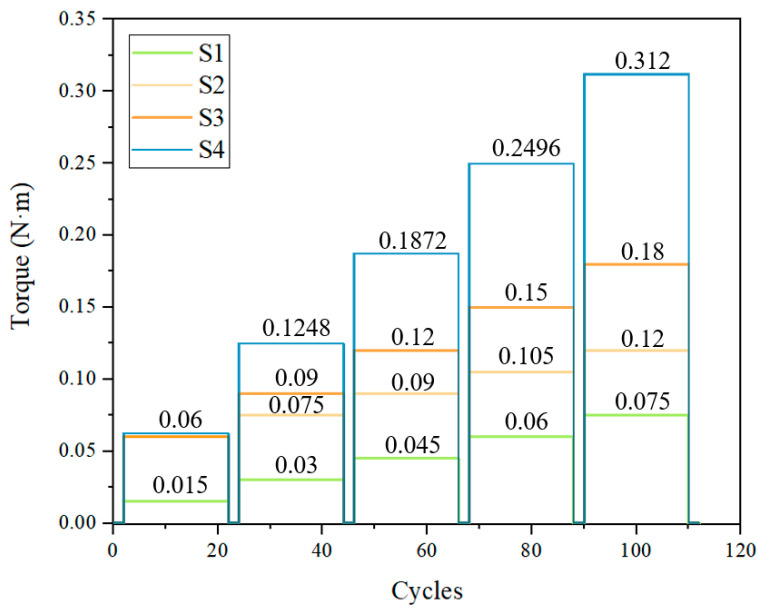
Torques used in the experiment.

Experiments of constant-torque thermal cycling were conducted. In the experiment, the SMAMS was loaded and thermally cycled. The upper and lower cycle temperatures are 120 °C and 25 °C, respectively. At each loading value, the torque was held constant, while the tube was thermally cycled 20 times. After every 20 cycles, each SMAMS was thermally cycled under no load to evaluate the two-way shape memory effect (TWSME), or lack thereof, as shown in Figure 9.

## 5. Results and Discussions

**Figure 10:**
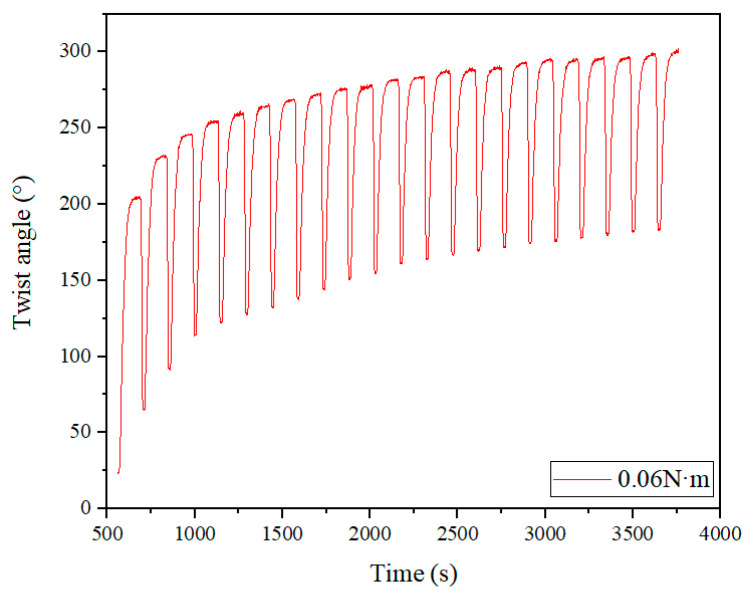
Twist angle–time curve of the SMAMS S2.

The twist angle reflects the actuating capability of the SMAMS. The representative twist angle over time (cycles) for the SMAMS S2 under a torque of 0.06 N·m can be seen in Figure 10. This figure shows that, upon increasing the number of cycles, the twist angle of the SMAMS increases quickly and then gradually reaches a relatively steady state. Specifically, the maximum twist angle of the SMAMS in the first cycle is 204.5°, and after 20 cycles, this angle increases to 300.7°. This variety is caused by the accumulation of plastic deformation during the actuating process. As the number of cycles rises, the accumulation of plastic deformation increases and then tends to be stable. It is worth noting that this variety is not caused by heat accumulation. As can be seen in the figure, the twist angle finally tends to be stable during the cooling process in each cycle. Therefore, there is no heat accumulation in each cycle. It can also be seen in this figure that, upon increasing the number of cycles, the actuating twist angle of the SMAMS decreases slightly. Specifically, the actuating twist angle of the SMAMS in the first cycle is 140°, and after 20 cycles, this value decreases to 117.6°. The results show that the actuating capacity of the SMAMS reduces slowly and gradually stabilizes as the number of cycles increases. The actuating angles of the SMAMS, both here and below, reflect the shape memory effect of the SMA. The inner and outer surfaces of the SMAMS undergo martensitic transformation under the load and then return to the initial position under a high temperature.

**Figure 11:**
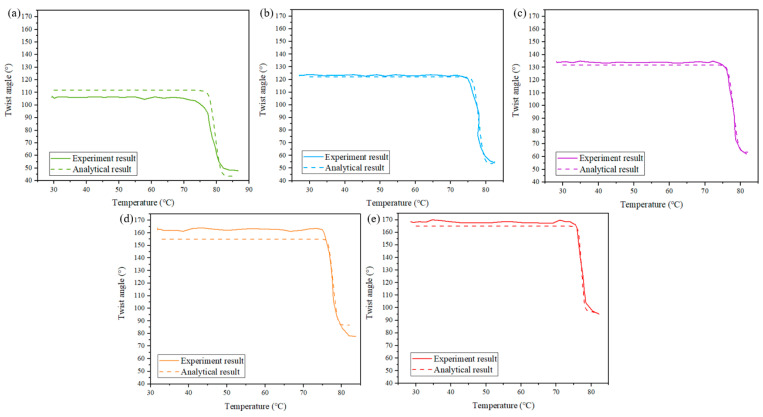
Experimental and analytical modeling results for SMAMS S2 subjected to applied torques corresponding to the 20th cycle. Torques are (**a**) 0.06 N·m, (**b**) 0.09 N·m, (**c**) 0.12 N·m, (**d**) 0.15 N·m and (**e**) 0.18 N·m.

Figure 11 shows the experimental and analytical modeling results for SMAMS S2 subjected to different applied torques corresponding to the 20th cycle. The SMAMS S2 is used as an example in this analysis, and similar results can be obtained for SMAMSs of different sizes. Because the analytical model does not take into account the evolution of the plastic strain in SMA, the 20th cycle after the stability of SMA performance in the test was used as the comparative analysis. The solid lines represent the twist angle of the SMAMS at different applied torques. As can be seen from the figure, the twist angles of the SMAMS vary from 59.9° at a torque of 0.06 N·m to 91.2° at a torque of 0.18 N·m. The dotted lines represent the analytical results. Both results were found to provide similar trends for all parameter variations including temperature and applied torques. The value of errors between experiment results and analytical results at 0.06, 0.09, 0.12, 0.15 and 0.18 N·m are 5.5%, 1.1%, 1.85%, 5.4% and 2%, respectively. The error is within the allowable range, so the experimental results verify the correctness of the above analytical model.

**Figure 12:**
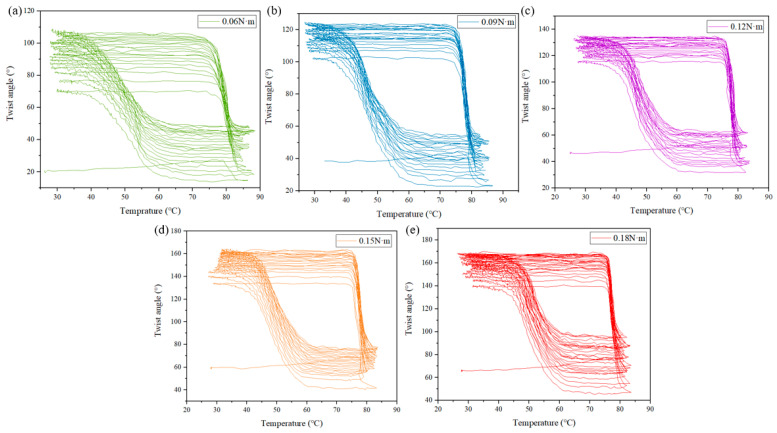
The twist angle–temperature responses of S3 subjected to 20 constant-torque thermal cycles. Torques are (**a**) 0.06 N·m, (**b**) 0.09 N·m, (**c**) 0.12 N·m, (**d**) 0.15 N·m and (**e**) 0.18 N·m.

The relationship between the twist angle and temperature is important to analyze the properties of the SMAMS, such as phase transition temperature and dimensional stability. Taking the S3 as an example, the twist angle–temperature curves of S3 subjected to 20 constant-torque thermal cycles are shown in Figure 12.

**Figure 13:**
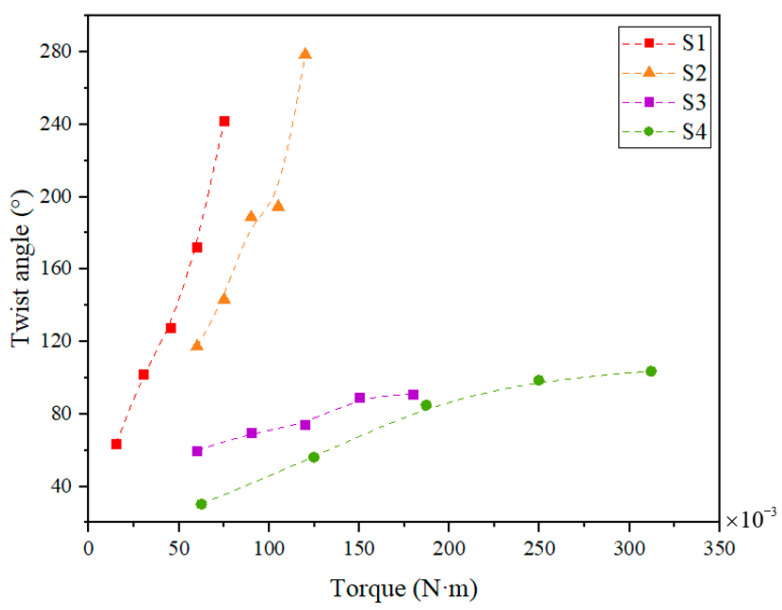
Summary of the twist angle as a function of the applied torque.

Figure 13 shows a summary of the twist angle as a function of the applied torque. In this figure, in all geometries, the twist angle of SMAMSs gradually increases with the rise of the applied torque. Specifically, the twist angle of S1 ranges from 63.5° at a torque of 0.015 N·m to 241.9° at 0.075 N·m; S2 ranges from 117.3° at a torque of 0.06 N·m to 278.5° at 0.12 N·m; S3 ranges from 59.9° at a torque of 0.06 N·m to 91.2° at 0.18 N·m; S4 ranges from 30.2° at a torque of 0.0624 N·m to 103.6° at 0.312 N·m. In addition, the parameters of SMAMSs also have a significant influence on the twist angle, which will be analyzed in the following part.

**Figure 14:**
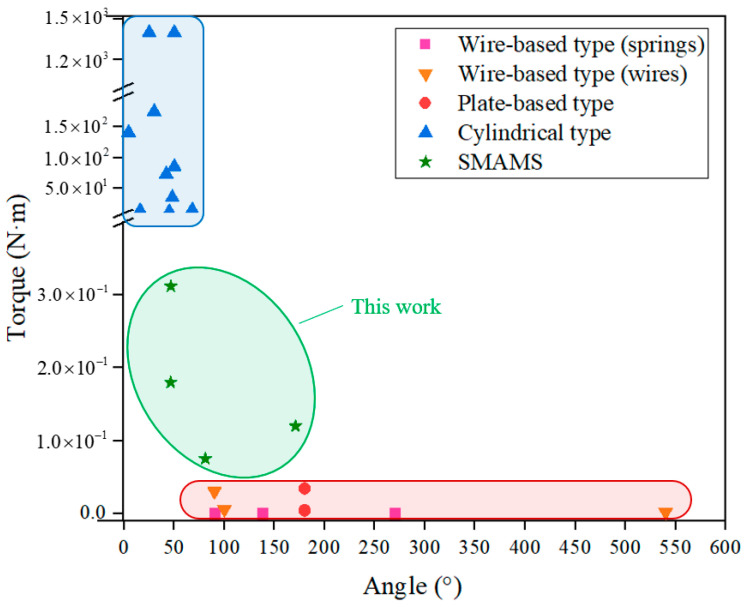
Comparisons between the SMAMSs and other types of SMA rotary actuators, ie. wire-based-type actuators [15,16,17,18,19,39], plate-based-type actuators [20,21,24] and cylindrical-type actuators [25,26,27,28,31,32].

Figure 14 shows the comparisons between the SMAMS (green) and other types of SMA rotary actuators, i.e., wire-based-type actuators [15,16,17,18,19,39] (red), plate-based-type actuators [20,21,24] (red) and cylindrical-type actuators [25,26,27,28,31,32] (blue). For rotary actuators including both wire-based-type actuators and plate-based-type actuators, the capability in the red dotted line area indicates that they can generate larger twist angles and bear smaller torques. However, for cylindrical type actuators, the capability appears to be the opposite. They can generate smaller twist angles and bear larger torques. The SMAMS presented in this work (green) have a balanced performance. They can be designed to have a relatively large torsion angle and to bear a relatively large torque.

**Figure 15:**
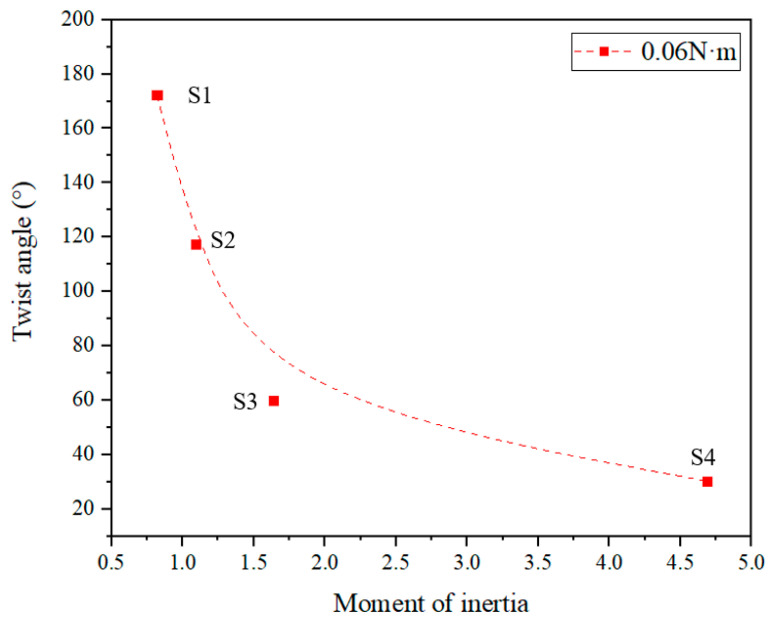
The relationship between the twist angle and the moment of inertia of SMAMSs.

To better demonstrate the effect of geometry on the twist angle, we used the moment of inertia to characterize the geometry of SMAMSs and obtained the relationship between the twist angle and the moment of inertia of SMAMSs, as shown in Figure 15.

Figure 15 shows the twist angle of SMAMSs reduces as the moment of inertia increases under the torque of 0.06 N·m. Specifically, the moments of inertia of S1, S2, S3 and S4 are 0.82, 1.09, 1.64 and 4.69, respectively. Their corresponding twist angles are 185.3, 171.8, 58.7 and 29.8, respectively. In general, the SMAMSs with a larger moment of inertia can withstand a larger load.

**Figure 16:**
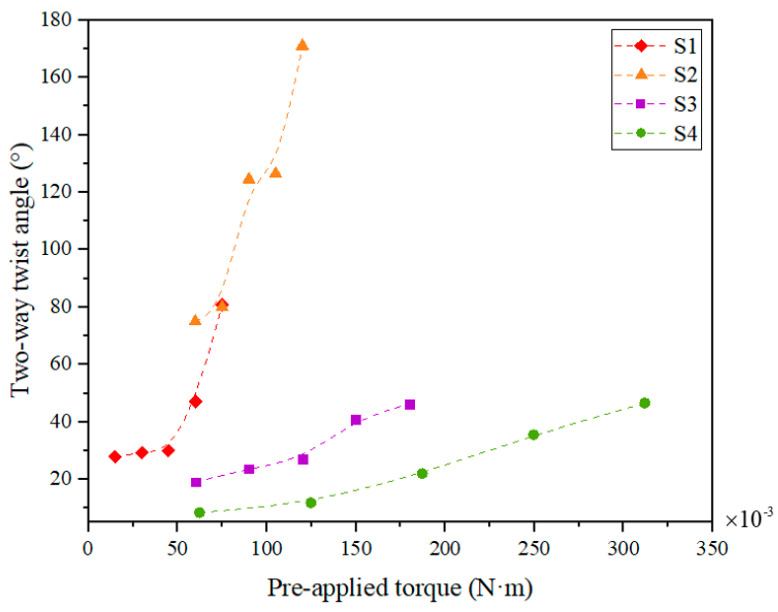
Two-way twist angle vs. the pre-applied torque.

Similarly, the two-way twist angle of SMAMSs in Figure 16 follows a consistent trend with the twist angle in Figure 14. Specifically, the two-way twist angle of S1 ranges from 27.9° at a pre-applied torque of 0.015 N·m to 80.9° at 0.075 N·m; S2 ranges from 75° at a pre-applied torque of 0.06 N·m to 171° at 0.120 N·m; S3 ranges from 19.1° at a pre-applied torque of 0.06 N·m to 46.3° at 0.18 N·m; S4 ranges from 8.4° at a pre-applied torque of 0.0624 N·m to 46.6° at 0.312 N·m. The two-way effect is known to be driven by internal stresses. The smaller SMAMSs exhibit higher stress gradients across the coil wall thickness, resulting in a higher two-way effect.

## 6. Conclusions

This paper has proposed a novel SMAMS. An analytical model was developed to design the SMAMS, and a numerical model was established to analyze the stress and mechanical responses of the flexure of the SMAMS and other rotary actuators during deformation. These results laid the foundation for the later experiments. Four different sizes of SMAMSs were fabricated, and constant-torque thermal cycling experiments were designed and conducted. Based on the above results, the main conclusions can be summarized as follows:

(1) The SMAMS can be designed to have a larger twist angle than the tube and to bear a larger torque than the spring of the circular cross-section, provided that the inner and outer diameter remains unchanged, which fills the gap between wire-based-type rotary actuators and cylindrical-type rotary actuators.
(2) The twist angle of the SMAMS gradually increases with the applied torque. Specifically, the twist angle of S2 ranges from 117.3° at a torque of 0.06 N·m to 278.5° at 0.12 N·m; S4 ranges from 30.2° at a torque of 0.0624 N·m to 103.6° at 0.312 N·m.
(3) The twist angle of SMAMSs decreases as the moment of inertia increases under a constant applied torque.
(4) The two-way twist angle of SMAMSs decreases as the pre-applied torque increases. Specifically, the two-way twist angle of S2 ranges from 75° at a pre-applied torque of 0.06 N·m to 171° at 0.12 N·m; S4 ranges from 8.4° at a pre-applied torque of 0.0624 N·m to 46.6° at 0.312 N·m.

## Figures and Tables

**Table 1:** SMA material properties.

Property	Symbol	Value
Young’s modulus (austenite-phase)	*D*_a_	67 GPa
Young’s modulus (martensite-phase)	*D*_m_	26.3 GPa
Maximum residual strain	*ε*_L_	0.038
Thermal expansion coefficient	*Θ*	0.55 MPa/°C
Austenite-phase finish temperature	*A*_f_	82 °C
Austenite-phase start temperature	*A*_s_	77 °C
Martensite-phase finish temperature	*M*_f_	40 °C
Martensite-phase start temperature	*M*_s_	58 °C
Stress influence coefficient for austenite	*C*_A_	13.8 MPa/°C

**Table 2:** Geometric parameters.

Parameter	Symbol	S1	S2	S3	S4
Outer diameter	*D*_o_	30 mm	30 mm	30 mm	31.2 mm
Inner diameter	*D*_i_	26.6 mm	26.6 mm	26.6 mm	25.8 mm
Cross section ⊥ to spring axis	*b*	1.6 mm	1.6 mm	1.6 mm	2.6 mm
Cross section ∥ to spring axis	*h*	2.4 mm	3.2 mm	4.8 mm	3.2 mm
No. of active coils	*n*	5	5	5	5
Pitch	*p*	6 mm	6 mm	6 mm	6 mm
Overall length	*l*	60 mm	60 mm	60 mm	60 mm
Spring index	-	17.68	17.68	17.68	10.96

## Data Availability

The data presented in this study are available on request from the corresponding author.
